# Endoscopic submucosal dissection including the papilla for a duodenal adenoma using a high-pressure injection knife

**DOI:** 10.1055/a-2779-7554

**Published:** 2026-02-05

**Authors:** Yohei Ikenoyama, Reiko Yamada, Misaki Nakamura, Yasuhiko Hamada, Noriyuki Horiki, Hayato Nakagawa

**Affiliations:** 1 220937 Department of Gastroenterology and Hepatology, Mie University Hospital, Mie, Japan

**Fig. 1:**
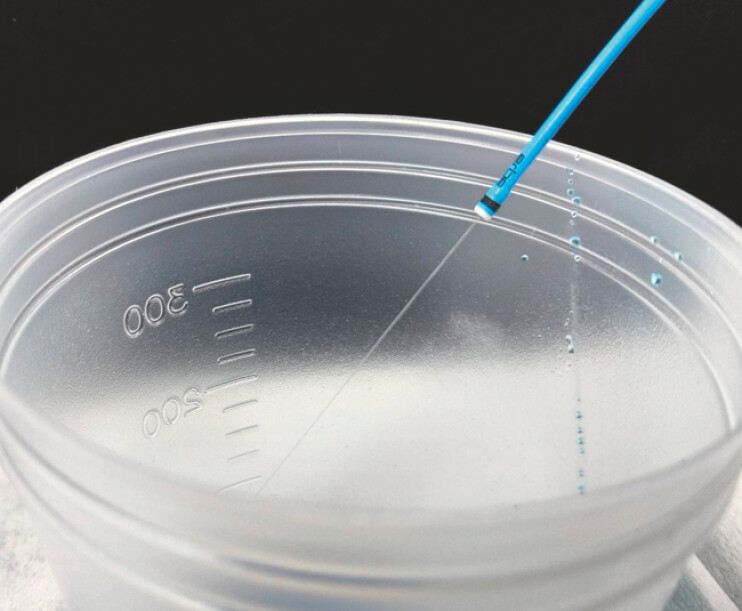
The novel HybridKnife allows both submucosal injection and dissection with a single device.

Endoscopic submucosal dissection (ESD) in the duodenum is technically demanding because of its complex anatomy and the high risk of perforation 1 2 . ESD involving the major duodenal papilla (ESDIP) is particularly challenging and is performed only in selected high-volume centers 3 . Resection of the papilla exposes the mucosal defect to bile and pancreatic juice, increasing the risk of bleeding and perforation. Therefore, a device that enables safe and swift resection is needed. The recently developed new-generation HybridKnife flex (Erbe, Tübingen, Germany) combines both submucosal injection and dissection within a single device, eliminating the need for device exchange ( Fig. 1 ). We report the first case of ESDIP performed using the HybridKnife flex 4 .

**Fig. 2:**
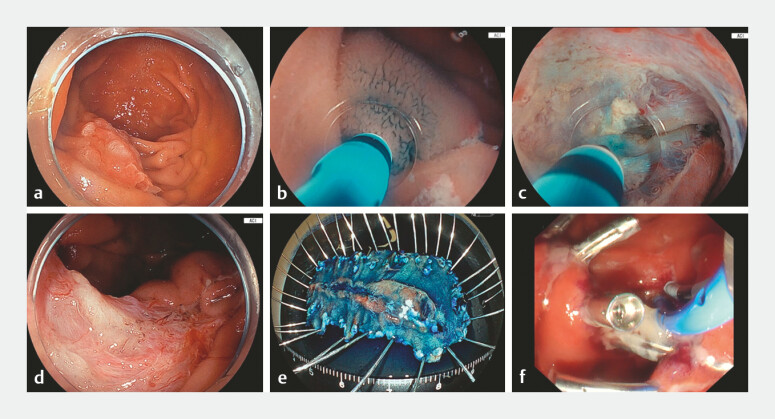
Endoscopic images. **a** The lesion was presented as a 30-mm tumor identified on the inner wall of the descending duodenum, involving the major duodenal papilla. **b** Adequate submucosal injection was easily obtained. **c** The major duodenal papilla could be safely resected with additional submucosal injections. **d** En block resection was achieved. **e** Pancreaticobiliary drainage was performed just after resection. **f** Histopathological evaluation confirmed the curative resection.

A 77-year-old woman was found to have a laterally spreading 30-mm tumor on the inner wall of the descending duodenum involving the major papilla ( Fig. 2 , Video 1 ). Biopsy confirmed an adenoma. ESDIP was performed using the HybridKnife flex T-Type (1.5 mm), which provides excellent tissue purchase and strong hemostatic capability when used with the needleless high-pressure water-jet system (ERBEJET 2). A therapeutic endoscope (EG-840T; Fujifilm, Tokyo, Japan) with an EP-8000 light source (Fujifilm, Tokyo, Japan) was used. Electrosurgical settings included EndoCut U mode (Effect 1, Duration 4, Interval 2) and PreciseSect mode (Effect 3.5) with a VIO 3 electrosurgical generator (Erbe, Tübingen, Germany).

Endoscopic submucosal dissection including the papilla for a duodenal adenoma using a highpressure injection knife.Video 1

High-pressure submucosal injection of saline mixed with indigo carmine through the device tip provided sufficient submucosal elevation for safe incision and dissection. The needleless system allowed instant, controlled injection and enabled efficient dissection without injection-related bleeding. The papillary portion and the remaining lesion were removed safely, with a total procedure time of 59 minutes. Pancreaticobiliary drainage was performed immediately after resection, and histopathology confirmed curative R0 resection.

The HybridKnife flex may facilitate safe and efficient ESD for technically challenging duodenal papillary lesions.

Endoscopy_UCTN_Code_TTT_1AO_2AG_3AD
